# Metformin Alters Locomotor and Cognitive Function and Brain Metabolism in Normoglycemic Mice

**DOI:** 10.14336/AD.2019.0120

**Published:** 2019-10-01

**Authors:** Wenjun Li, Kiran Chaudhari, Ritu Shetty, Ali Winters, Xiaofei Gao, Zeping Hu, Woo-Ping Ge, Nathalie Sumien, Michael Forster, Ran Liu, Shao-Hua Yang

**Affiliations:** ^1^Department of Pharmacology and Neuroscience University of North Texas Health Science Centre, Fort Worth, TX76107, USA.; ^2^Children's Research Institute, Department of Paediatrics, University of Texas, Southwestern Medical Center, Dallas, TX 75390, USA; ^3^Department of Neuroscience, Department of Neurology & Neurotherapeutics, University of Texas, Southwestern Medical Center, Dallas, TX 75390, USA

**Keywords:** metformin, metabolism, diabetes, AMP-activated protein kinase, cognition

## Abstract

Metformin is currently the most effective treatment for type-2 diabetes. The beneficial actions of metformin have been found even beyond diabetes management and it has been considered as one of the most promising drugs that could potentially slow down aging. Surprisingly, the effect of metformin on brain function and metabolism has been less explored given that brain almost exclusively uses glucose as substrate for energy metabolism. We determined the effect of metformin on locomotor and cognitive function in normoglycemic mice. Metformin enhanced locomotor and balance performance, while induced anxiolytic effect and impaired cognitive function upon chronic treatment. We conducted *in vitro* assays and metabolomics analysis in mice to evaluate metformin’s action on the brain metabolism. Metformin decreased ATP level and activated AMPK pathway in mouse hippocampus. Metformin inhibited oxidative phosphorylation and elevated glycolysis by inhibiting mitochondrial glycerol-3-phosphate dehydrogenase (mGPDH) *in vitro* at therapeutic doses. In summary, our study demonstrated that chronic metformin treatment affects brain bioenergetics with compound effects on locomotor and cognitive brain function in non-diabetic mice.

Metformin is currently the most effective treatment for type-2 diabetes (T2D) with well-established anti-hyperglycemia action [1]. Metformin has been found to be protective against diabetic angiopathy, cardiomyopathy, and nephropathy in T2D patients and to reduce the incidence of diabetes in high-risk individuals. The beneficial actions of metformin may even extend beyond diabetes management, including antidepressant effects and reducing the risk of cancer [2-4]. Furthermore, metformin has been found to improve health and extend lifespan in normoglycemic mice [5, 6], and is considered as one of the most promising drugs that could potentially slow down aging [7, 8].

T2D patients are at an increasing risk of developing neurological disorders. While the beneficial action of metformin against the peripheral diabetic complications has been well established, the action of metformin on CNS has been less studied and conflicting information exists about the beneficial versus adverse effects of metformin on brain function [9, 10]. On the positive side, metformin treatment has been shown to enhance neurogenesis and spatial learning in female C57/129J mice at a dose of 200 mg/kg per day [11], attenuate oxidative stress in brain of Goto-Kakizaki male rats at a dose of 100 mg/kg per day [12], and reduce tau phosphorylation in mice at a dosage relevant to high clinical dosage (5 mg/ml drinking water) [13]. Longitudinal clinical studies have indicated that metformin may reduce the risk of cognitive decline in diabetic patients [2, 14]. Metformin treatment improved cognitive function and produce antidepressant effects in diabetic patients with depression [3]. On the negative side, metformin did not attenuate cognitive deficits in high-fat-diet-fed male Wistar rats at a dosage of 144 mg/kg in diet [15] and leptin-resistant male db/db mice at a dosage of 200 mg/kg/day [16]. Metformin has been demonstrated to upregulate BACE1 transcription and increase Aβ production in C57BL/6 mice at a dose of 2 mg/ml in drinking water [17], increase memory dysfunction in males but is protective in females using a mouse model of AD [18]. Metformin increased amyloid β aggregation in mice [17, 19, 20]. Metformin treatment resulted in a significant worsening of performance in Morris water maze (MWM) test for spatial learning and memory [21]. Large population-based case-control studies have indicated that chronic metformin was associated with impaired cognitive performance [22] and greater risk of neurodegenerative diseases including AD [23, 24]. A recent systematic review and meta-analysis of 14 out of 862 published clinical studies indicated that metformin use was associated with reduced risk of dementia in patients with diabetes but not support the use of metformin for the prevention of dementia in no diabetes patients [25].

The mammalian brain is characterized by high metabolic activity with tight regulatory mechanisms to ensure adequate energy substrates delivery in register with neuronal activity. Surprisingly, the effect of metformin on brain metabolism has not been explored given that brain almost exclusively uses glucose as substrate for energy metabolism under physiological condition [9, 10]. Determining the effect of metformin on brain bioenergetics may provide important insight for the action of metformin on brain function. In the current study, we determined the effect of metformin on locomotor and cognitive function in normoglycemic mice using extensive behavioral tests. We further determined the effect of metformin on brain metabolism and the potential underlying mechanisms.

## MATERIALS AND METHODS

### Reagents and Cells

Antibodies for pAMPKα, AMPKα, pTSC2, TSC2, p-Raptor, Raptor, pERK, ERK were purchased from Cell Signaling Technology. Metformin, glycerol 3-phosphate (G3P), LCMS grade water, and methanol for metabolites analysis were purchased from Sigma. Postnatal primary astrocytes and primary neurons were prepared as described in our previous studies from postnatal day 0-1 C57BL/6J mouse pups [26, 27]. Primary astrocytes were maintained in DMEM medium with 10% FBS and was shaken overnight to get rid of microglia. Primary astrocytes were used at passage 1 and 2. Primary neurons were cultured in Neurobasal medium with B27 supplement. AraC (1 µM) were added to inhibit astrocyte proliferation and removed before experiments.

### Animal and metformin treatment

Procedures for animal treatment were approved by the University of North Texas Health Science Center Institutional Animal Care and Use Committee (IACUC). Four-month-old adult male C57BL/6J mice were purchased from the Jackson Laboratory, housed in clear polycarbonate cages at 23 ± 1°C under a 12-h light/dark cycle, and fed *ad libitum*. Metformin was added to drinking water (2 mg/ml) and the mice were treated for indicated time. After treatment, mice were euthanized and perfused with cold saline to remove blood. Brains were then dissected for analysis.

### Behavioral tests

Mice were randomly assigned to the experimental groups. (1) Control: fed normal water; (2) metformin: fed water fortified with 2 mg/ml metformin. The mice were on their respective treatments for 4 weeks prior to and throughout behavioral assessments for a total of 12 weeks. Food and water intake were measured daily at the end of the behavior tests at 9 am over a period of 5 days. All values presented were an average of all the 5 days of these measurements. Body weights were measured before the start of behavior tests and at the end of behavior tests daily for 5 days. Values expressed are an average of 5-day measurements. Blood glucose levels were measured at the end of the behavior tests. The behavioral tests were conducted in the following order: open field exploration (locomotor activity), coordinated running (motor learning and maximum motor performance), muscle strength in wire suspension, balance in elevated bridge walk, spatial learning and memory (Morris water maze), active avoidance (T-maze), and anxiety assessment (elevated zero maze).

Spontaneous locomotor activity was measured using a Digiscan apparatus (Omnitech Electronics, model RXYZCM-16) as described previously [28] [29]. Each mouse was placed in a clear acrylic test cage (40.5×40.5×30.5 cm) that was surrounded by a metal frame lined with photocells for 4 consecutive sessions, each session with 4-minute in duration for a total of 16 minutes. During a 16-minute period, movements in the horizontal plane and vertical plane were detected by the photocells and processed by a software program to yield different variables describing distance, vertical, and spatial components of spontaneous activity in the apparatus.

An accelerated rotarod test was used to measure motor learning and maximum running performance as described previously using a motor-driven treadmill (Omnitech Electronics, Model # AIO411RRT525M) [29]. On a given trial, the mouse was placed on the 3-cm diameter nylon cylinder at a height of 35-cm above a padded surface, which began rotating with increasing speed until the animal fell on the padded surface. The ability of the mice to improve running performance was assessed in a series of training sessions (2 per day), each consisting of 4 trials at 10-minute intervals. The training sessions continued until the running performance (the average latency to fall from the cylinder) failed to show improvement over 3 consecutive sessions. The treatment and control groups were compared for their average latency to fall on the first 7 sessions and for the final session on which each mouse had reached its maximum performance.

Wire Suspension test for muscle strength and musculoskeletal reflex was conducted as described previously [29]. Wire suspension test was conducted over 4 consecutive daily sessions (twice a day). In each test, the mouse was allowed to grip a horizontal wire with the front paws when suspended 27-cm above a padded surface. The latency to tread (reaches the wire with their hind legs) and the latency to fall were recorded and averaged over 4 consecutive daily sessions (2 trials /day).

Bridge walking test was conducted as described previously [29]. Each mouse was tested for the latency to fall or reach a safe platform after being placed on 1 of 4 acrylic bridges, each mounted 45-cm above a padded surface. The bridges differed in diameter (small or large) and shape (round or square), providing 4 levels of difficulty. Each bridge was presented 3 times, and the measure of performance was the latency to fall, either examined as the average latency to fall (of 3 trials) for each bridge individually, or a single overall mean representing the average latency to fall from all 4 types of bridges.

Spatial learning and memory were measured using an Morris Water Maze test as described previously [30]. On a given trial, the mouse was allowed to swim in a tank filled with opacified water maintained at 24 ± 1 °C. The mouse was able to escape the water by reaching a hidden platform (1.5 cm below the surface of the water). A computerized tracking system recorded various measures such as path length and swimming speed (Any-maze; Stoelting Co., Wood Dale, IL, USA). The test consisted of 3 phases: (1) Pre-training phase: the tank was covered by a black curtain to hide surrounding visual cues. The mouse learned the components of swimming and climbing onto a platform using a straight alley that had a platform at one end. The mouse was allowed to swim until they reached the platform, or a maximum of 60 seconds had elapsed. The mouse received 2 sessions consisting of 5 trials with an inter-trial interval of 5 minutes. (2) Acquisition phase: the black curtain was removed, and the mice were tested for their ability to locate a hidden platform using spatial cues around the room. Each daily session consisted of 5 trials, at 2-minute intervals, during which the mouse had to swim to the platform from 1 of 4 different starting points in the tank. The mouse was allowed to swim until they reached the platform, or a maximum of 90 seconds had elapsed. Testing was conducted over 9 sessions (Tuesday-Friday and Monday-Friday). On sessions 2, 4, 5, 7, and 9, a probe trial was conducted as the fifth trial during which the platform was submerged to a depth that prevented the mouse from climbing onto it. The platform was raised after 30 seconds and the trial was ended when the mouse successfully located it. (3) Retention phase: one 60-second probe trial session was conducted 1 week after the ninth session of the previous phase. Path length (distance taken to reach the platform) over sessions was used as the primary measure of performance. The path-independent swim speed was calculated by dividing distance by the latency to reach the platform. Latency (time taken to reach the platform) over sessions was used as an additional measure of performance. On probe trial, spatial bias for the platform location was evaluated in terms of the percentage of time spent within a 40-cm diameter annulus surrounding the platform location.

A T-maze constructed of acrylic (black for the sides and clear on the top) was utilized for the discriminated avoidance task. The maze was divided into 3 compartments: a start box, a stem, and 2 goal arms. The maze rested on a grid floor wired to deliver 0.69-mA scrambled shock to the feet of mice. The test consisted of 3 sessions separated by 1 hour. On each training trial, the mouse was placed in the start box and the start door was removed to signal the beginning of the trial. Shock was initiated 5 seconds after the opening of the start door if the mouse had not entered the correct goal arm or immediately upon entry into the incorrect arm. Training is continued at 1-minute intervals until the mouse had met the criterion of a correct avoidance (defined as running directly to the correct arm within 5 seconds) on 4 of the last 5 training trials of which the last 2 must be within 5 seconds. Two measures were considered to show the ability of the mouse to learn the discrimination and avoidance components of the task. Their ability to learn was considered inversely proportional to the number of trials required to reach the avoidance criterion aforementioned and the number of trials required to reach the discrimination criterion (4 out 5 correct turns regardless of the time taken).

To measure anxiety, an elevated zero maze test was conducted using a zero-maze elevated 3 feet above the floor in a dimly lit test room (60 W) consisting of 2 arms opened to the room and 2 arms enclosed such that the floor is not visible. A computerized tracking system was used to monitor the position of the mouse in the maze (Any-maze). The mouse was positioned at the junction of open and close arms facing an open arm and were given 5 minutes to explore the maze. The amount of time the mouse spent in the closed vs. open arms was recorded.

### Oxygen consumption rate measurement and ATP assay

Oxygen consumption rate (OCR) and extracellular acidification rate (ECAR) were monitored using a Seahorse Bioscience XF24 Extracellular Flux Analyzer. Primary astrocytes were seeded at 50,000 per well and cultured overnight before treatment in a XF24 seahorse plate. Primary neurons were prepared in the XF24 plate and cultured for more than 10 days before assay. Rotenone, FCCP, and oligomycin were diluted into XF24 media and loaded into the accompanying cartridge to achieve final concentrations of 2 μM, 1 μM, and 1 μg/ml, respectively. Injections of the drugs into the medium occurred at the time points specified. Each cycle was set as: mix for 3 minutes, delay for 2 minutes and then measure for 3 minutes. For the G3P seahorse assay, there was a 10-minute delay instead of 2-minute delay in each cycle. After Seahorse analysis, each well was washed with PBS to remove cellular debris and protein concentration was determined. The OCR was measured and normalized by protein in each wells. ATP concentration was measured using a commercial kit (Invitrogen, Eugene, OR, USA). ATP concentrations were normalized to the protein concentration measured with a protein assay kit (Thermo Scientific, Rockford, IL, USA). ATP concentration of the hippocampus was measured from the tissue lysate for metabolomic analysis and normalized to protein concentration of each sample. ATP level was presented as percentage of the mean value of the control group.

### Mitochondrial complex I assay and mGPDH activity assay

Brain mitochondria were isolated from 4-month old male C57BL/6J mice. Briefly, brain was removed placed in ice-cold mitochondrial isolation buffer containing 210 mM mannitol, 70 mM sucrose, 5 mM HEPES, and 1 mM EDTA, pH 7.4. The brain was homogenized immediately, and the mitochondrial fraction was isolated by differential centrifugation as described previously [31]. Complex I activity assay was performed as described in previous studies with minor modifications [32, 33]. Mitochondria were sonicated 3 times for 30 seconds on low power to break apart mitochondrial membranes and expose the individual complexes of the electron transport chain. For complex I activity assay, mitochondrial membrane fractions were added to 50 mM phosphate buffer (pH = 7.4) containing 2 mM MgCl2, 2 mM KCN, antimycin A (2 mM) and 150 µM NADH. Complex I activity was monitored by adding NADH to the mix with or without rotenone (6 µM). Coenzyme Q1 (CoQ1) was added to the mixture and kinetic change in NADH absorbance at 340 nm were monitored with a plate reader. Complex I activity normalized to control levels for statistical analysis. Assay for mGPDH activity was conducted as described in previous study [34]. Briefly, fresh brain mitochondria isolation was used for assay in reaction buffer (10?mM Tris-HCl, 200 μM glycerol-3-phosphate, 50?μM cytochrome c, 25?μM sodium azide, 1?mM EDTA, 50?mM KCl). Cytochrome c gain of absorbance at 550 nm was measure using a plate reader. Metformin was incubated with mitochondria for 10 minutes before the assay.

### Metabolomic analysis

Hippocampus was dissected on a cold plate and frozen in liquid nitrogen. Tissue was then homogenized in 80% methanol (made with LCMS grade methanol and water) on ice using a sonicator homogenizer. A small amount was taken for protein assay analysis. The rest samples were centrifuged at 15000 rpm and the supernatant was transferred to a new tube. After normalized using protein assay, specific volume of the supernatant was dried in a vacufuge concentrator at 30 ^o^C. The samples were then submitted to mass spectrometry analysis for metabolites.

### Statistical Analysis

All data were presented as mean ± S.E.M. The significance of differences among groups with one independent variable was determined by one-way ANOVA with a Tukey’s multiple comparisons test for planned comparisons between groups when significance was detected. The significance of differences among groups where two independent variables presented were determined by two-way ANOVA. P-value <0.05 (*) was considered significant. Functional performance of the mice on the behavioral tests was assessed using unpaired *t*-test with control and metformin as between-group factors. To understand the learning process over different session, repeated measure was employed. The α level was set at 0.05 for all analyses. The software used for the analyses was Graph Pad Prism V.7 (GraphPad Software, Inc. CA, USA).

**Figure 1.**
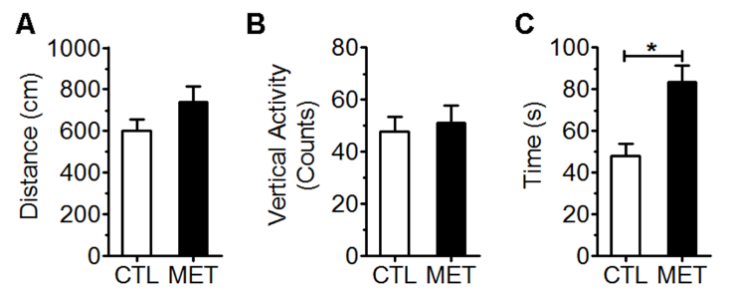
Effect of metformin treatment on spontaneous locomotor activity in young male normoglycemic C57BL/6J mice. The locomotor activity was measured in terms of total distance travelled in cm (A), vertical activity counts (B), and time spent in the center zone (C). CTL: control mice fed with drink water. MET: mice fed with drink water with metformin (2 mg/ml). Each value represents mean ± SEM, n=10. * p<0.05 vs control.

## RESULTS

### Metformin improved locomotor function

We determined the effect of chronic metformin treatment on locomotor function with clinically effective dose range. The final metformin dosage was calculated to be 181.8 mg/kg/day based on the daily water consumption, which is comparable to a human dose of 884.4 mg/day for 60 kg body weight. Metformin group has a slightly higher food intake compared to the controls although no statistical significance was found (*p*=0.2926) (data not shown). Metformin treatment had no effect on body weight and blood glucose level (*p*=0.0845) in young adult male mice (data not shown).

**Figure 2.**
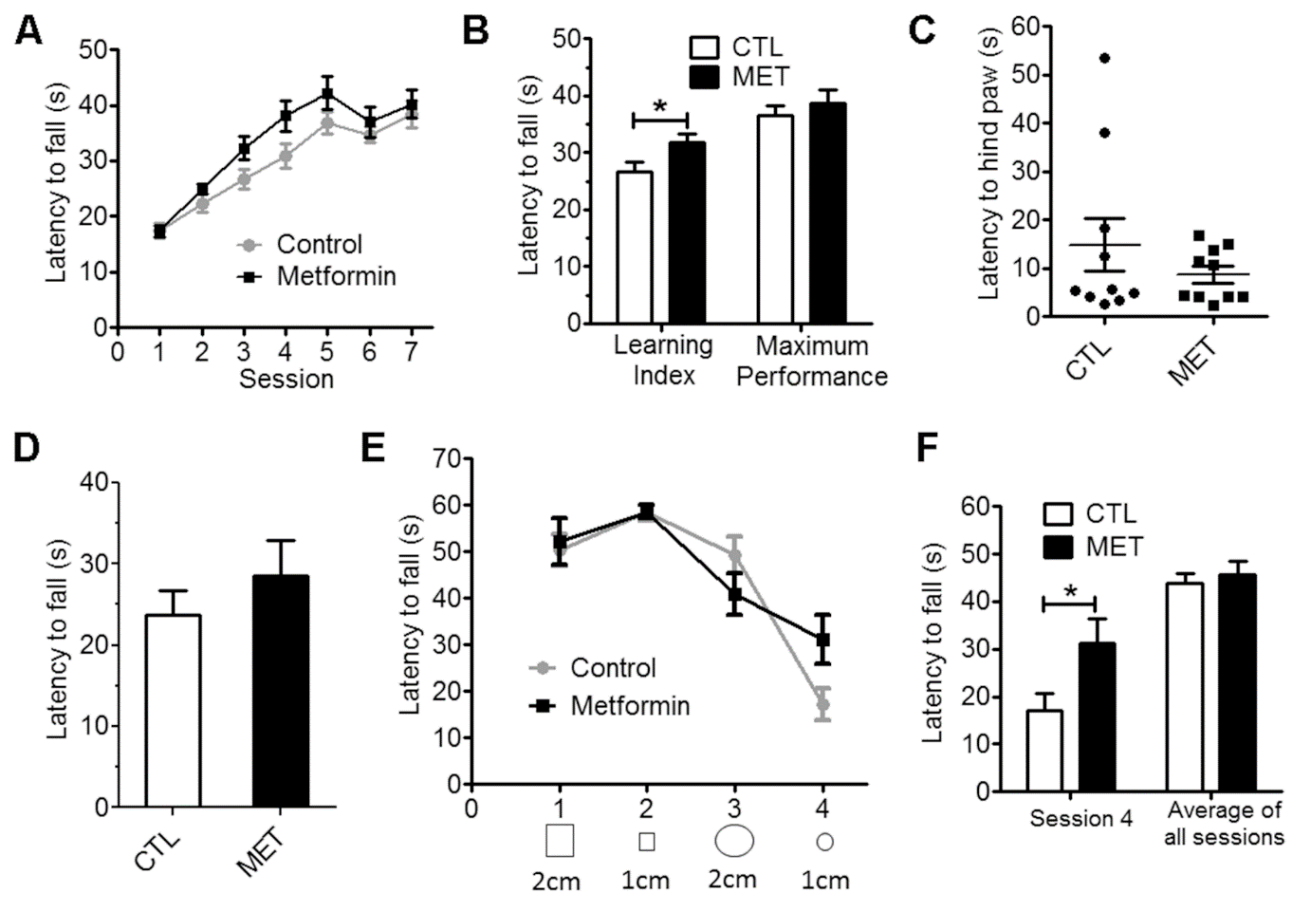
Metformin improved coordinated running and balance performance in young male normoglycemic mice. Session wise (A) and learning index and maximum performance (B) of rotarod test demonstrated metformin treatment improved coordinated running. Wire suspension test demonstrated that metformin did not affect motor reflex (C) and muscle strength (D). Bridge walking test demonstrated that metformin treatment improved balance performance (E & F). Session wise performance and (E) and session 4 and average of balance beam test (F) in bridge walking test. Each value represents mean ± SEM, n=10. * *p*<0.05 vs control.

No significant effect on spontaneous activity was found upon metformin treatment as measured by travel distance and rearing (Fig. 1A, *p*=0.157; Fig. 1B. *p*=0.697). However, metformin-treated mice spent significant more time in the center zone of an open field as compared with controls (Fig. 1C, *p*<0.01), suggesting an anxiety alleviating effect of metformin. Improved coordinated motor performance over sessions was observed in both control and metformin groups evidenced by a significant effect of the session (*p*<0.001) (Fig. 2A). In addition, a better motor performance was indicated in the metformin treatment group as compared with the control. Difference between treatment groups was more pronounced during the learning phase of the task (sessions 2 through 4) than when a plateau was reached (session 6 and 7). Metformin treatment had significantly longer latencies during the learning phase (p<0.05), but not the plateau phase (p=0.515), than the controls (Fig. 2B).

We determined the effect of metformin treatment on reflexive musculoskeletal responses using wire suspension test. Measurement of reflexes and strength was presented as latencies to tread using hind paw and fall. No significant effect of metformin treatment on the latency to hind paw was observed (Figure 2C). The metformin-treated group had a trend to have a longer latency to fall although no significance was reached (*p*=0.376) (Fig. 2D).

The effect of metformin on motor and balance function was assessed using bridge walk test over 4 consecutive days (Figure 2E & F). Metformin treatment had no effect on average bridge walking performance of all 4 sessions. However, metformin treatment significantly improved bridge walking performance in session 4, the most difficult bridge task, suggesting the chronic metformin treatment might enhance motor and balance performance (*p*<0.05) (Fig. 2E & F).

### Metformin treatment impaired cognitive function

**Figure 3.**
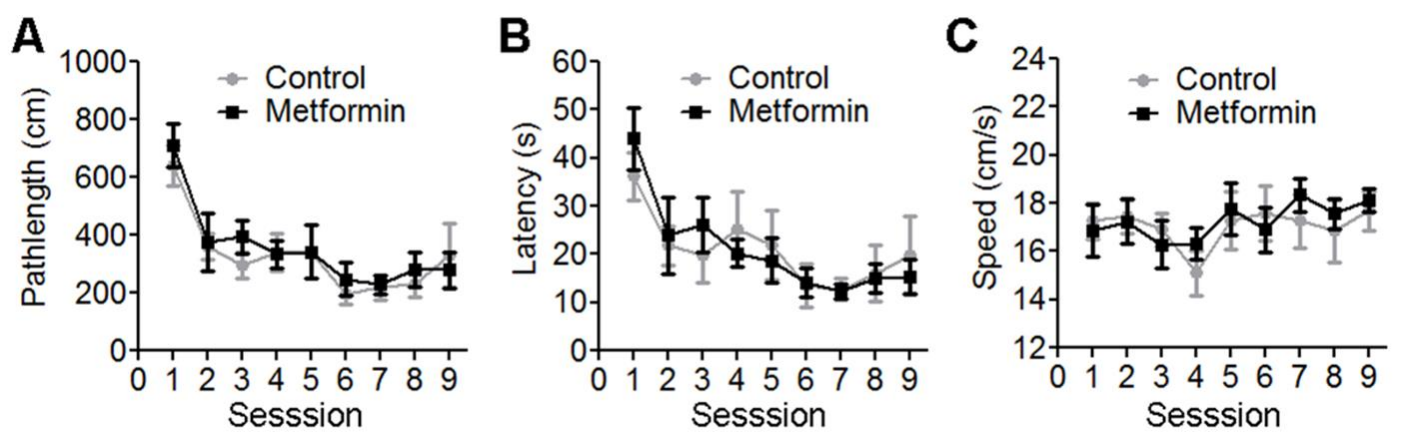
Metformin did not alter Morris water maze spatial learning in young male normoglycemic C57BL/6J mice. The data represents Morris water maze outcome over 9 sessions and shown as path length in cm (A), latency in seconds (B) and swim speed in cm/second (C). Across all sessions, there was no significant difference between control and metformin fed mice. Each value represents mean ± SEM, n=10.

**Figure 4.**
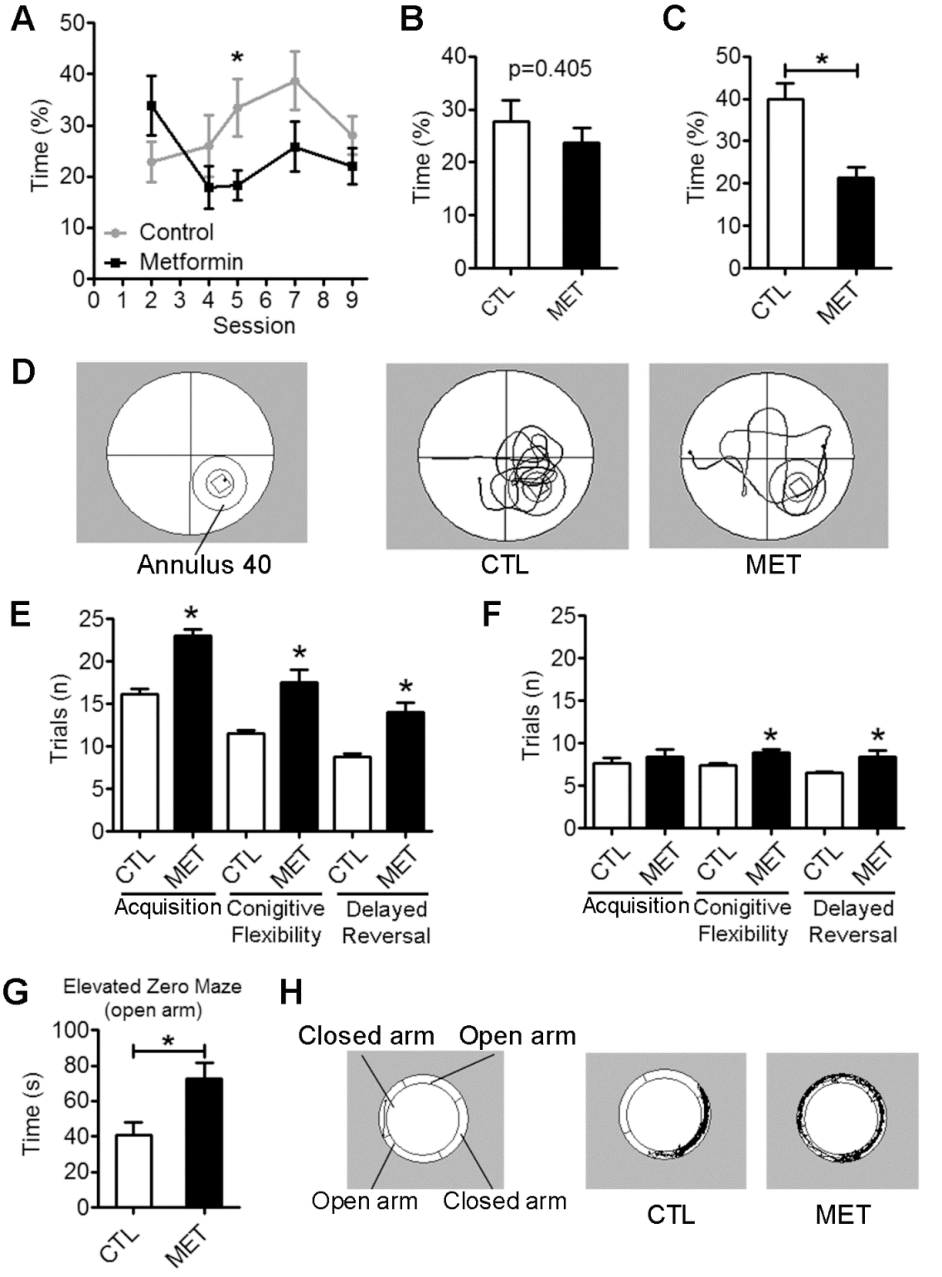
Metformin treatment impaired spatial memory function and decreased anxiety in young male normoglycemic mice. Morris water maze (MWM) test (A to D) demonstrated metformin treatment impaired delayed spatial memory: session wise time (%) (A) and average time (%) (B) spent in annulus 40 across probe trials 2,4,5,7 and 9. (C) Delayed spatial memory measured as time (%) spent in annulus 40 in session 10 in MWM test. (D) Representative path of control (CTL) and metformin (MET) treated mice in session 10 in MWM test. Discriminative avoidance test (E & F) demonstrated that metformin treatment impaired acquisition, cognitive flexibility and delayed reversal performance. Trials (n) to reach avoidance criterion (E) and trials (n) to reach discrimination criterion (F) in discriminative avoidance test. Elevated Zero maze test demonstrated that metformin treatment decreased anxiety. Time (s) spent in open arms (G) and representative path of CTL) and MET mice (H). Each value represents mean ± SEM, n=10. *p<0.05 compared to control.

The performance of the mice in learning the spatial swim maze task was assessed by path length and latency to reach the hidden platform. Mice in control and metformin treatment groups learned the task over session as observed with repeated measure ANOVA (*p*<0.001). Metformin treatment had no effect on swim speed (*p*>0.05) (Figure 3). Accuracy for spatial working memory was further measured by probe trial at sessions 2, 4, 5, 7, and 9. In addition, time spent in annulus 40, the virtual area of 40-cm diameter around the platform, was used as an indicator of spatial memory. Metformin-treated mice showed a trend to spend less time in annulus 40 area as compared to the controls, which reached significance for session 5 (Figure 4A, *p*<0.05). While the more composite average of all 5 probe trials for annulus 40 indicated no impact of metformin on spatial memory (Fig. 4B, *p*=0.405). Delayed spatial retention was tested 1 week after the last session in a single probe trial (session 10) for 60 seconds. All the mice retained the previously learned information. However, metformin treatment significantly impaired delayed spatial memory component of water maze when tested for time spent in annulus 40 (*p*<0.001) (Fig. 4C and D).

We determined the effect of metformin treatment on discriminated avoidance learning. The metformin-treated mice took 41.97%, 52.17%, and 59.09% more trials to reach the avoidance criterion than control mice in the acquisition, cognitive flexibility, and delayed reversal, respectively (*p*<0.01) (Fig. 4E). In discrimination testing, metformin-treated mice took 9.09%, 20.27%, and 29.23% more trials than control mice in the acquisition, cognitive flexibility (*p*<0.05), and delayed reversal (*p*<0.05), respectively (Fig. 4F). In elevated zero maze test, metformin-treated mice spent 77.68% more time in the open arm as compared with controls, suggesting an anxiolytic effect of metformin (*p*<0.05) (Fig. 4G & H).

### Metformin altered brain metabolism in vivo

**Figure 5.**
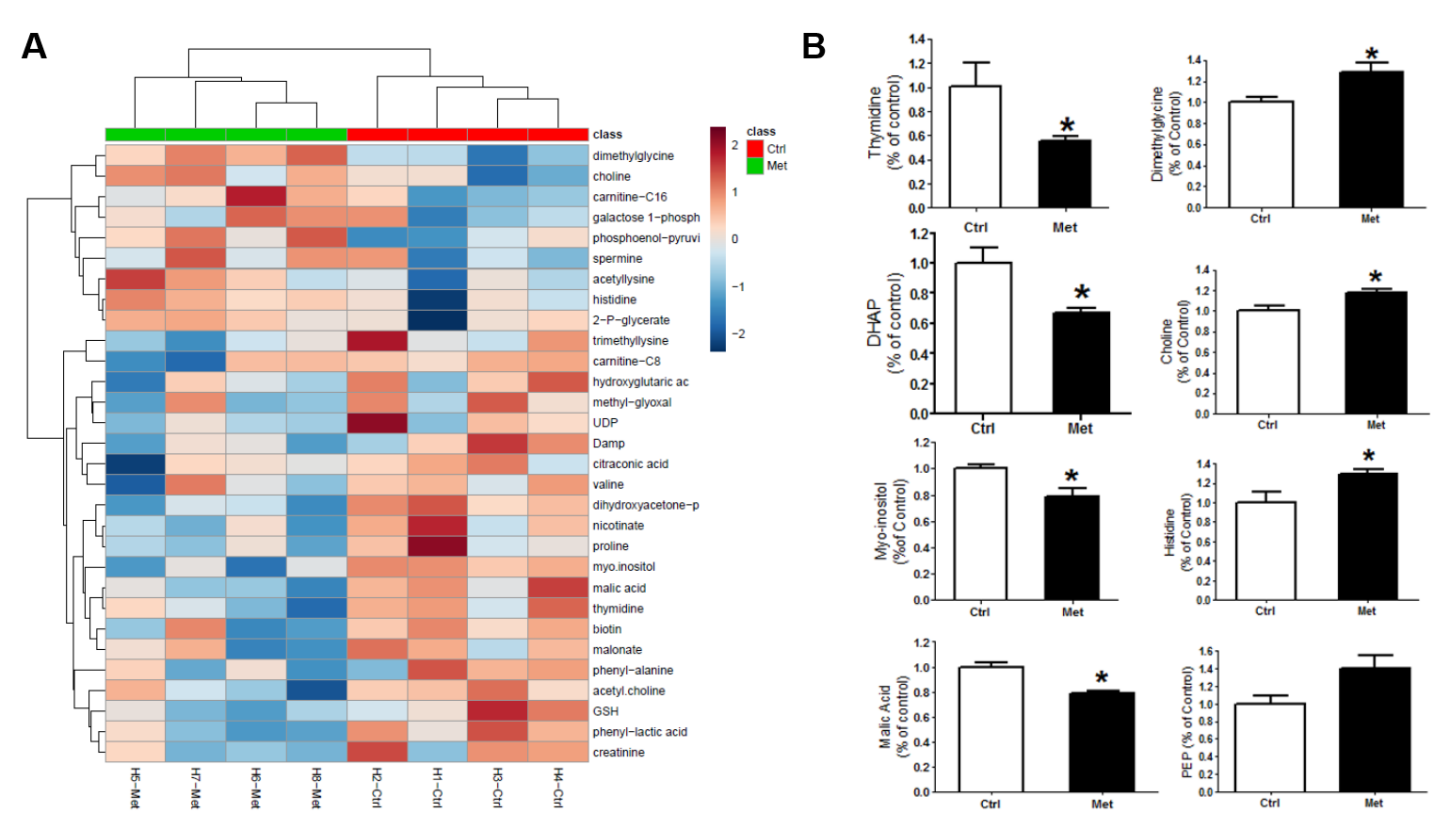
Metformin treatment altered metabolomic profile in hippocampus of young male normoglycemic mice. A) Heatmap of 145 metabolites (Supplementary Table) in mice hippocampus after 7-day treatment with metformin in drinking water (2mg/ml). B) Metformin treatment significantly increased levels of dimethylglycine, histidine, and choline in the hippocampus. Significant decreased levels of malic acid, thymidine, dihydroxyacetone-phosphate (DHAP), and myo-inositol were observed in the hippocampus of metformin-treated mice (* *p*<0.05) (n=4).

**Figure 6.**
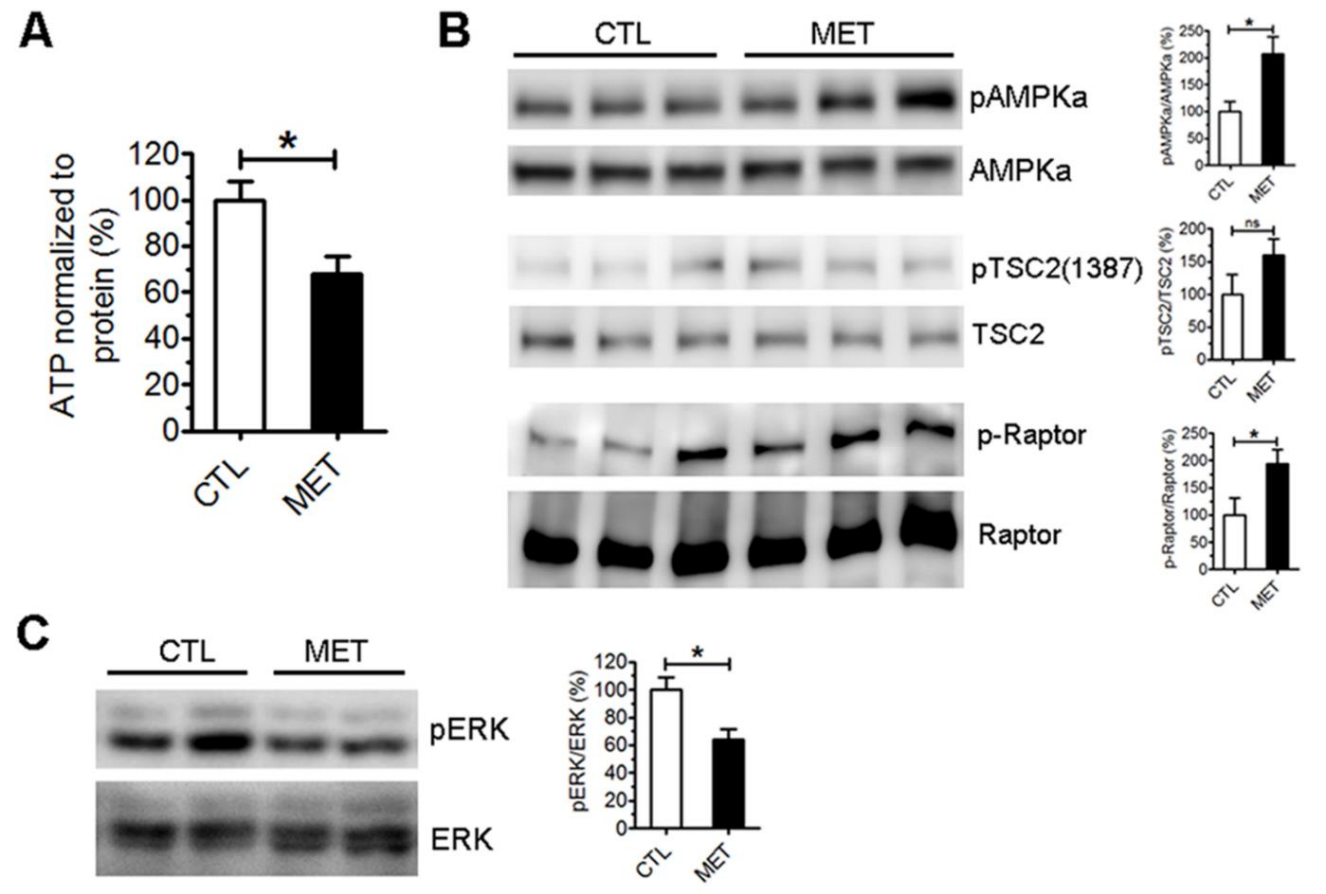
Metformin treatment decreased ATP level and activated AMPK in the hippocampus of young male normoglycemic mice. A) ATP assay of mouse hippocampus lysate after 7-day treatment with metformin. ATP level was normalized to protein concentration (n=4). B) Representative and quantitative analysis Western blots of total and phosphorylated AMPKα, TSC2 and Raptor in hippocampus of metformin treated (MET) and control (CTL) mice (n=4) (* *p*<0.05). C) Western blot analysis of phosphorylation of total and phosphorylated ERK in hippocampus of metformin treated (MET) and control (CTL) mice (n=4) (*, *p*<0.05).

To examine the effect of metformin on brain metabolism *in vivo*, a separated set of male C57BL/6J mice were administered with 2 mg/ml metformin in drinking water or control for 7 days (n=4 each group). Brains were harvested and dissected for unbiased metabolomic analysis. A clear alteration of metabolites levels was observed in the hippocampus after metformin treatment (Fig. 5A, supplementary Table 1). The levels of dimethylglycine, histidine, and choline were significantly elevated in the hippocampus upon metformin treatment (*p*<0.05) (Figure 5B). Significant decreased levels of malic acid, thymidine, dihydroxyacetone-phosphate (DHAP), and myo-inositol (*p*<0.05) were observed in the hippocampus of metformin-treated mice (Figure 5B). Metformin treatment significantly reduced ATP level in the hippocampus (Figure 6A). Activation of AMPK signaling was found in the hippocampus of metformin-treated mice evidenced by the increased phosphorylation of AMPKα and its downstream TSC2/Raptor (Figure 6B). In addition, significant decrease of ERK activation was observed after metformin treatment (Figure 6C).

### Clinically relevant dose of metformin reduced oxygen consumption rate and increased glycolysis in vitro

**Figure 7.**
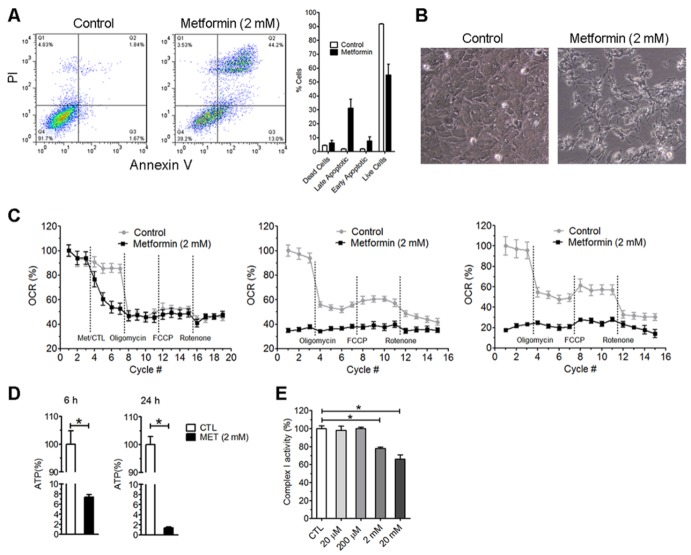
High dose metformin induces cell death and inhibits mitochondrial complex I. Primary astrocytes were treated with 2 mM metformin for 48 hours. A) Left panel, flow cytometer analysis after Annexin V and PI; right panel: percentage of cell in each state. B) Bright image of astrocytes after 48-hour metformin or control treatment. C) Oxygen consumption rate (OCR) of primary astrocytes immediately (left panel), at 6 (middle panel), or 24 hours (right panel) after metformin treatment or control (final concentration of metformin was 2 mM). D) ATP level after 6-hour and 24-hour treatment with 2 mM metformin. E. Complex I activity of mitochondria isolated from adult mice after incubation with different concentrations of metformin for 10 minutes. Metformin inhibited mitochondrial complex I activity at 2 and 20 mM. (* *p*<0.05).

We tested the effect of high dose metformin (2 mM) on viability of mouse primary astrocyte. A 48-hour treatment of mouse primary astrocytes with 2 mM metformin induced significant cell death. Double staining of PI and Annexin V suggest increased apoptotic and necrotic cells after metformin treatment (Figure 7A). Metformin treatment caused dramatic shrinkage of cell body (Figure 7B). We determined the effect of 2 mM metformin on oxygen consumption in primary astrocytes using Seahorse XF24 Extracellular Flux analyzer. We found that administration of high dose of metformin to astrocytes immediately reduced oxygen consumption rate (Figure 7C). Six-hour and 24-hour treatment further reduced oxygen consumption rate. Consistently, ATP production was significant reduced after 6-hour and 24-hour treatments (Figure 7D). We isolated brain mitochondria from adult mouse and analyzed the effect of metformin on brain mitochondria activity. We found that metformin at 2 mM and 20 mM inhibited complex I activity of the isolated brain mitochondria, while 20 and 200 µM had no effect on complex I activity (Figure 7E). Our results demonstrated that millimolar dose metformin inhibited brain mitochondria complex I activity and reduced oxidative phosphorylation, which could cause brain cell death.

**Figure 8.**
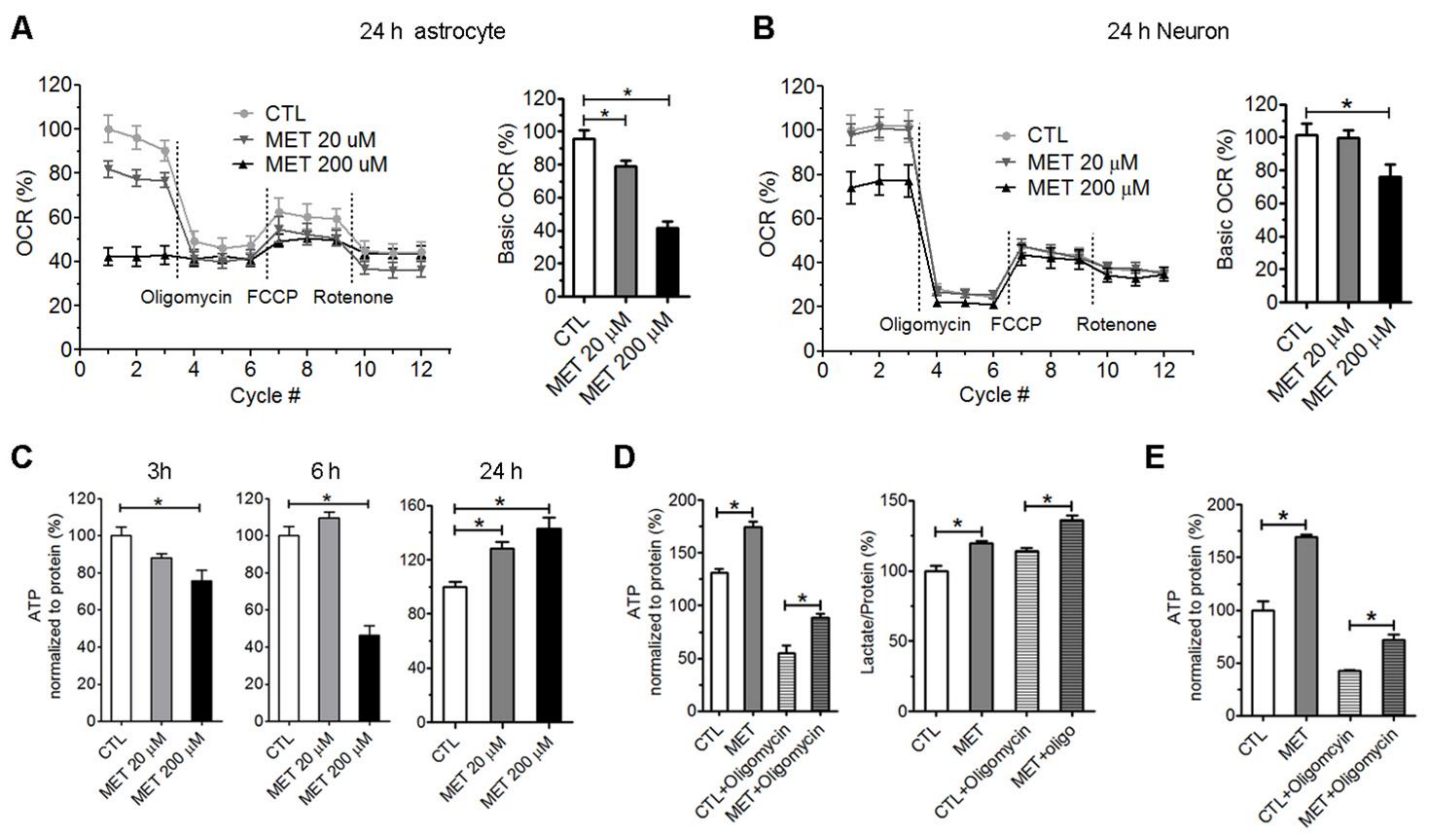
Prolonged treatment with clinically relevant concentrations of metformin inhibited oxygen consumption rate (OCR) and increased ATP production through enhancement of glycolysis pathway *in vitro*. Primary astrocytes (A) and primary neurons (B) were treated with metformin (20 µM and 200 µM) for 24 hours before seahorse extracellular flux analysis; bar graph indicated base OCR of each condition (n=6). C) ATP assay in primary astrocyte culture after treatment with 20 µM and 200 µM metformin for 3, 6 and 24 hours (n=6). D) Total ATP in primary astrocytes was increased after 24-hour treatment with 50 µM metformin. Astrocyte was treated with 10 mg/ml oligomycin for 2 hours to inhibit ATP production from mitochondrial oxidative phosphorylation. Lactate production was increased by metformin treatment (n=6). E) Total ATP (24-hour treatment) and ATP from glycolysis (2-hour treatment) in primary neurons were increased by 50 µM metformin treatment (n=6) (* *p*<0.05).

Plasma concentrations of metformin in patients and animals treated with therapeutic dose have been found to be between 10 to 50 µM [35, 36]. We found that acute treatment with 20 and 200 µM metformin had no effect on oxygen consumption rate (OCR) in primary astrocytes within 6 hours (data not shown). However, OCR was reduced to ~80% and 40% of baseline after 24-hour treatment of 20 and 200 µM metformin, respectively (Figure 8A). In primary neurons, 24-hour treatment of 20 µM metformin did no significantly reduce OCR, while 200 µM metformin reduced OCR to ~70% of baseline (Fig. 8B). These results suggest that clinically relevant doses of metformin could reduce oxygen consumption in primary astrocytes and primary neurons. Interestingly, primary astrocytes seem were more susceptible to metformin as compared with primary neurons.

We further determined the effect of therapeutic doses metformin on ATP production in primary astrocytes. ATP production was significantly reduced after 3- and 6-hour treatment of metformin at 200 μM. Surprisingly, a significant increase of ATP production was observed after 24-hour metformin treatment (Figure 8C). ATP is generated by both glycolysis and mitochondrial oxidative phosphorylation in astrocytes. We speculated that the increase of ATP production upon metformin treatment was likely attributed to glycolysis. Primary astrocytes were treated with 50 μM metformin for 24-hour and then subjected to ATP and lactate assay. Oligomycin was added to inhibit oxidative phosphorylation at 2-hour before ATP and lactate analysis. As we expected, metformin increased ATP production even with the inhibition of mitochondrial oxidative phosphorylation. Significant increases in lactate production were observed after metformin treatment independent to oligomycin (Figure 8D). Consistently, in primary neurons, significant increases of ATP production were found after 24-hour treatment with 50 µM metformin with and without oligomycin (Figure 8E). These results suggest that metformin could inhibit oxidative phosphorylation and increase glycolysis in primary astrocytes and neurons, resulting in an increase of ATP production through glycolytic pathway.

### Metformin inhibited brain mitochondria glyceral-3-phosphate dehydrogenase (mGPDH)

**Figure 9.**
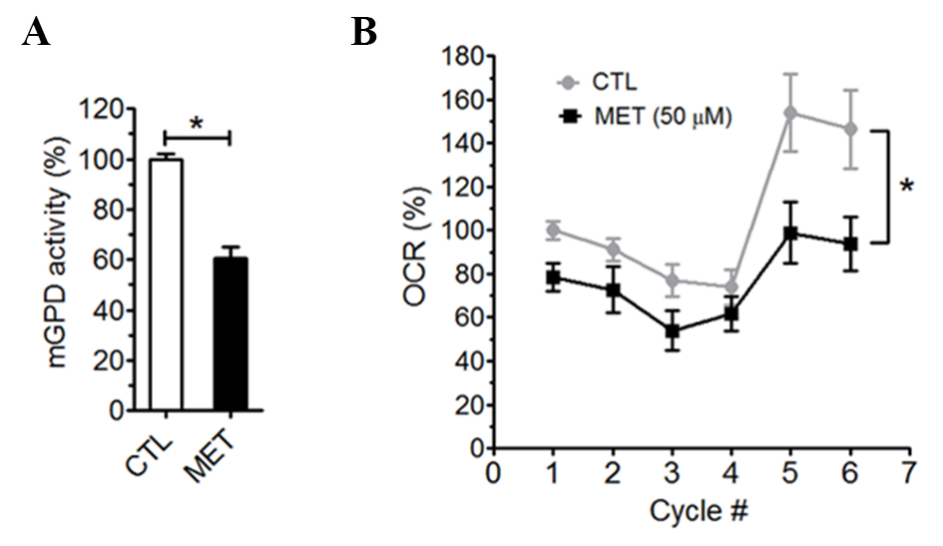
Metformin inhibited brain mGPDH. A) mGPDH activity in mitochondria isolated from mouse brain was inhibited by 50 µM metformin (n=4). B) Metformin inhibited G3P induced oxygen consumption in primary astrocytes in seahorse extracellular flux assay (n=8).

A recent study has demonstrated that therapeutic dose metformin could inhibit mGPDH [34]. Inhibition of mGPDH blocks transportation of electron from glycolysis-derived NADH into mitochondria, which may lead to the reduction of mitochondrial oxidative phosphorylation. We isolated mitochondria from adult mice brains to determine the effect of metformin on mGPDH activity. Significant inhibition of mGPDH activity was found upon treatment of 50 µM metformin (Figure 9A). mGPDH catalyzes the irreversible oxidation of glycerol 3-phosphate (G3P) to dihydroxyacetone phosphate (DHAP), transferring 2 electrons to mitochondrial electron transport chain. Indeed, in seahorse assay without glucose, G3P was used as a substrate for oxidative phosphorylation when mitochondrial complex I was inhibited by rotenone. Treatment of 50 µM metformin blocked oxygen consumption induced by G3P in primary astrocytes (Figure 9B). These data indicate that therapeutic doses of metformin inhibit brain mitochondrial oxidative phosphorylation through the inhibition of mGPDH.

## DISCUSSION

We have identified 3 important founding in the current studies. First, we found that chronic metformin treatment at a clinically relevant dose had a complex effect on locomotor and cognitive function in normoglycemic male mice. Second, the negative effect of metformin on cognitive function was associated with an activation of AMPK signaling and change of metabolomic profile at the hippocampus. Third, the effect of metformin on inhibition of oxidative phosphorylation and enhancement of glycolysis was likely mediated through the inhibition of mitochondrial glycerol-3-phosphate dehydrogenase (mGPDH).

The current studies demonstrated that chronic metformin treatment altered brain function evidenced by the extensive behavioral test battery. Interestingly, metformin seems to have a distinctive effect on locomotor and cognitive function upon chronic treatment with a clinically relevant dose. On one hand, metformin treatment improved coordinated motor and bridge walking performance. On the other hand, metformin treatment had a negative effect on cognitive function evidenced by the impairment in delayed spatial memory and discriminated avoidance learning. The observed effect of metformin on locomotor and cognitive function was subtle while statistically significant. Nonetheless, the dose used in our study was low as comparable to a human dose of less than 1,000 mg/day. We predict that metformin treatment of a maintenance dose at 2,000 mg daily in human may have a more dramatic effect on locomotor and cognitive functions.

The brain is by far the most expensive organ in term of energy expenditure in the whole body characterized by high metabolic activity with fine regulatory mechanisms to ensure adequate energy substrates supply in register with brain activity. Under the normal physiological condition, adult brain almost exclusively uses glucose as substrate for energy metabolism [37, 38]. The action of metformin on glucose metabolism has been extensively studied in the peripheral system and there has been convincing data that place energy metabolism at the center of its antidiabetic action. The action of metformin on AMPK signaling, a key energy sensor and regulator, has been well established in many organs and cell types including brain [39-41]. Activation of AMPK has been found to be the primary anti-hyperglycemic action of metformin [40]. It has been further proposed that activation of AMPK was attributed to the inhibition of mitochondrial complex I and reduction of ATP production upon metformin treatment at the concentrations that may not be able to achieve with therapeutic dose *in vivo* [34-36, 42, 43]. The activation of AMPK by metformin is likely mediated by LKB1-independent mechanisms that may involve the inhibition of mitochondrial complex I and AMP deaminase [44-46]. More recently, metformin has been found to inhibit mitochondrial glyceral-3-phosphate dehydrogenase (mGPDH) within therapeutic dose range [34].

Metformin’s effect on cell metabolism has been explored using different concentrations. Most *in vitro* metformin studies have used doses more than 1 mM [47-49], which is much higher than the concentrations of metformin after the therapeutic dose *in vivo*. Plasma concentration of metformin in human diabetic patients has been found to be around 2.7 mg/L (14 µM). In rodents, plasma concentration of metformin was 10 to 40 µM after oral administration [35, 36]. After chronic treatment with metformin at a daily dose of 300 mg/kg, metformin concentration was 44 µM and 7.3 nmol/g in the cerebral spinal fluid (CSF) and hippocampus, respectively [36]. We determined the effect of metformin in primary astrocyte and neuron cultures using both high and low therapeutic concentrations. We found that 2 mM metformin immediately inhibited oxygen consumption rate in primary astrocytes, abolished oxygen consumption and reduced ATP production after 24-hour treatment. In isolation brain mitochondria, we found that 2 mM, not 200 µM, metformin inhibited mitochondrial complex I activity.

We further determined the effect of metformin on brain cell metabolism using clinically relevant concentrations. We found that that treatment of 20 µM or 200 µM metformin did not change oxygen consumption rate immediately. On the other hand, prolonged treatment with metformin decreased oxygen consumption rate in primary astrocytes. Metformin at 200 µM but not 20 µM decreased oxygen consumption rate in primary neuron cultures. Surprisingly, we found that 24-hour treatment increased ATP level, while oxygen consumption rate was reduced. When oxidative phosphorylation-dependent ATP production was inhibited by oligomycin, metformin still significantly increased the ATP production, suggesting a glycolysis-dependent mechanism.

We speculated that the bioenergetic action of metformin is mediated by the inhibition of mGPDH. mGPDH plays a key role in the glycerol-phosphate shuttle, which regenerates NAD+ from NADH by reducing of enzyme-bound flavin adenine dinucleotide (FAD) to FADH2 and contribute electrons to mitochondrial oxidative phosphorylation. Inhibition of mGPDH leads to accumulation of cytosolic NADH. We expected that NADH accumulated in the cytosol could react with pyruvate to generate lactate. Indeed, lactate production was significantly increased in primary astrocyte cultures upon metformin treatment at 50 µM. Consistent with the *in vitro* results, chronic metformin treatment at a clinically relevant dose indeed altered brain metabolism evidenced by our unbiased metabolomic analysis. In agreement with the mGPDH inhibition, decreased the level of DHAP and increased the level of G3P were observed in the hippocampus after metformin treatment. Both our *in vitro* and *in vivo* studies indicated that metformin, potentially via inhibition of mGPDH, reduces oxidative phosphorylation and increased glycolysis. Interestingly, a contradict effect on ATP production *in vitro* and *in vivo* was observed upon chronic treatment with metformin within therapeutic relevant dose. Chronic metformin treatment increased ATP production in primary astrocyte and neuron cultures while decreased ATP level at the hippocampus *in vivo*. We expected that the availability of glucose contributes to the discrepancy of ATP production upon chronic metformin treatment between in primary cultures and *in vivo* studies. Astrocytes and neurons were supplied with excessive glucose as a substrate for bioenergetics in primary cultures while brain has limited glucose supply controlled by regional CBF and glucose transporters in the blood brain barrier. The reduction of ATP level upon chronic metformin treatment indicated that the action of metformin in glycolytic ATP production is not able to compensate its inhibition of mitochondrial oxidative phosphorylation. Consistent with the decrease of ATP production *in vivo*, chronic metformin treatment altered major metabolic signals. A significant increase of AMPK activation was observed in the hippocampus by metformin treatment evidenced by the increase of pAMPK and its downstream TSC2 phosphorylation.

In summary, we determined the effect of metformin on brain function and bioenergetics. Our study indicated that metformin, at the clinically relevant therapeutic concentration/dose, inhibits mGPDH, reprograms brain bioenergetics from highly efficient mitochondrial oxidative phosphorylation to inefficient glycolysis pathway, reduces ATP production rate, activates AMPK signaling, hence, impact brain functions. Interestingly, chronic metformin treatment had complex effects on locomotor and cognitive function in non-diabetic mice. Metformin enhanced locomotor and balance performance. On the other hand, metformin treatment induced anxiolytic effect and impaired cognitive function upon chronic treatment. In the current study, the effect of metformin was only tested in young adult male mice. Further studies are needed to determine the metformin’s action in middle-age mice, including both male and female, which are more clinically relevant. Nonetheless, our study with other studies proposed careful re-consideration of metformin doses for translational research. The effect of clinically relevant therapeutic dose of metformin on brain bioenergetics and function revealed here may provide insights for the future study of metformin in neurological disorders.

## Supplementary Materials

The Supplemenantry data can be found online at: www.aginganddisease.org/EN/10.14336/AD.2019.0120
