# Supplementary material for: Metformin Alters Locomotor and Cognitive Function and Brain Metabolism in Normoglycemic Mice
Source: Aging Dis. 2019 Oct 1;10(5):949–63. doi: 10.14336/AD.2019.0120 (PMC6764722; doi:10.14336/AD.2019.0120)
Supplement: Supplementary file 1 — The Supplemenantry data can be found online at: www.aginganddisease.org/EN/10.14336/AD.2019.0120 [file AD-10-5-949-s.pdf]

SUPPLEMENTARY DATA

Metformin Alters Locomotor and Cognitive Function and Brain Metabolism in Normoglycemic Mice

Wenjun Li[1,#], Kiran Chaudhari[1,#], Ritu Shetty[1], Ali Winters[1], Xiaofei Gao[2], Zeping Hu[2], Woo-Ping Ge[2,3], Nathalie Sumien[1], Michael Forster[1], Ran Liu[1], Shao-Hua Yang[1, *]

[1]Department of Pharmacology and Neuroscience University of North Texas Health Science Centre, Fort Worth, TX76107, USA.
[2]Children's Research Institute, Department of Paediatrics, University of Texas, Southwestern Medical Center, Dallas, TX 75390, USA
[3]Department of Neuroscience, Department of Neurology & Neurotherapeutics, University of Texas, Southwestern Medical Center, Dallas, TX 75390, USA

© 2019. Li W et al. Published online at http://www.aginganddisease.org/EN/10.14336/AD.2019.0120

SUPPLEMENTARY DATA

Supplementary Table 1. Impact of metformin treatment on metabolic profile at the hippocampus of normoglycemic mice.

Sample Name	Hippo-ctrl AVERAGE	hippo-metformin AVERAGE	Drug/ctrl H	Ctrl/drug H	t-test t-test
spermine	1.64E-04	3.54E-04	2.156131022	0.463793707	0.227466634
phosphoenol-pyruvic acid	1.29E-03	1.82E-03	1.414367893	0.707029624	0.05931152
2,3-diphospho-D-glyceric acid	3.47E-04	4.87E-04	1.403402461	0.712553974	0.165901818
spermidine	4.53E-04	6.30E-04	1.39127516	0.718765079	0.42377625
propionylcholine	8.59E-03	1.14E-02	1.325685781	0.754326564	0.663678307
2-deoxycytidine	3.27E-05	4.28E-05	1.310998608	0.762777317	0.174797133
homoserine	3.91E-03	5.10E-03	1.303170197	0.767359476	0.171362532
histidine	3.21E-03	4.16E-03	1.297328184	0.770814981	0.046713114
carnitine.C8	2.70E-04	3.50E-04	1.296163702	0.771507487	0.358895248
2-P-glycerate	3.90E-03	5.02E-03	1.285767523	0.777745574	0.113463694
dimethylglycine	2.32E-03	2.98E-03	1.284976986	0.778224055	0.030372444
cadaverine	6.26E-05	7.87E-05	1.258063851	0.794872215	0.205386055
carnitine.C12	5.08E-05	6.33E-05	1.24653682	0.802222593	0.593773593
1-methyl-nicotinamide	2.07E-04	2.58E-04	1.245362098	0.802979312	0.414322086
GSSG	1.44E-04	1.77E-04	1.234196643	0.810243656	0.184868561
sn-glycero3-phosphate	1.36E-03	1.68E-03	1.230306282	0.812805734	0.379801987
carnitine.C6	6.06E-04	7.45E-04	1.228674227	0.813885388	0.236566121
fructose-1,6-biphosphate	2.40E-02	2.93E-02	1.221161656	0.818892401	0.199140532
xanthosine 5-monophosphate	3.64E-04	4.41E-04	1.210971105	0.825783535	0.15436797
galactose 1-phosphate	1.28E-03	1.53E-03	1.202096114	0.831880237	0.166287212
glutamine	8.94E-03	1.07E-02	1.19921344	0.833879914	0.107952894
UMP	1.71E-03	2.05E-03	1.198907134	0.83409296	0.109751771
UDP.glucuronic acid	3.74E-05	4.48E-05	1.197255155	0.835243845	0.400027598
adenosine 5-diphosphoribose	7.93E-03	9.47E-03	1.193659543	0.837759817	0.056400622
VitminB-3	4.46E-03	5.31E-03	1.192064859	0.838880529	0.287868072
choline	1.66E-02	1.95E-02	1.171653038	0.853494992	0.032699342
GMP	1.19E-03	1.39E-03	1.171505465	0.853602505	0.238788231
cystathionine	1.90E-04	2.22E-04	1.166146502	0.857525189	0.532551007
inosine	3.23E-04	3.76E-04	1.161847007	0.860698521	0.718579325
uridine	1.52E-03	1.76E-03	1.159268529	0.862612911	0.5437162
cytidine	1.76E-02	2.04E-02	1.157802685	0.863705028	0.490575505
guanine	7.78E-04	8.96E-04	1.151412221	0.868498685	0.377757066
carnitine.C5	9.13E-04	1.05E-03	1.14733313	0.871586442	0.598181246
carnitine.C4	9.31E-04	1.06E-03	1.140125425	0.877096483	0.682059284
IMP	1.24E-03	1.41E-03	1.139375367	0.877673881	0.248928919
CMP	7.47E-03	8.46E-03	1.132854462	0.882725922	0.252674166

© 2019. Li W et al. Published online at http://www.aginganddisease.org/EN/10.14336/AD.2019.0120

SUPPLEMENTARY DATA

allantoin	3.70E-05	4.19E-05	1.132052371	0.883351359	0.675381979
pipecolic acid	8.71E-03	9.84E-03	1.129627091	0.885247891	0.134112973
lysine	1.10E-02	1.24E-02	1.129140972	0.885629009	0.340882189
ribose 5-phosphate	9.15E-04	1.03E-03	1.12776423	0.886710159	0.549024532
taurine	8.72E-02	9.84E-02	1.127756042	0.886716597	0.07760147
methionine sulfoxide	2.11E-04	2.37E-04	1.121762764	0.891454086	0.621462357
phenyl-lactic acid	1.85E-04	2.07E-04	1.118533507	0.894027755	0.330326977
nicotinamide	1.29E-02	1.43E-02	1.105430316	0.904625091	0.484427056
G6P-F6P	2.08E-03	2.30E-03	1.104738946	0.905191225	0.545885659
thiamine	2.55E-04	2.81E-04	1.104711142	0.905214007	0.421431207
betaine	3.89E-03	4.25E-03	1.092362679	0.915446874	0.576153862
S.methyl.5.thioadenosine	3.77E-03	4.11E-03	1.090285082	0.917191308	0.592953794
tetrahydrobiopterin	5.86E-05	6.37E-05	1.087976746	0.919137292	0.338359598
asparagine	2.88E-03	3.13E-03	1.085919857	0.92087827	0.375501641
hypoxanthine	2.62E-03	2.85E-03	1.083992396	0.922515696	0.823787896
uracil	1.10E-04	1.19E-04	1.082418558	0.923857035	0.833791738
7.methylguanosine	4.13E-05	4.47E-05	1.081110893	0.924974493	0.584644107
N-acetyl-D-glucosamine 1-phosphate	3.76E-04	4.04E-04	1.076644435	0.928811748	0.821586839
1-methyl-histidine	9.16E-04	9.85E-04	1.07517951	0.930077248	0.709923813
carnitine	1.72E-02	1.83E-02	1.066133508	0.937968831	0.495311279
adenosine	6.07E-03	6.45E-03	1.061681017	0.941902496	0.878782246
acetyllysine	2.53E-03	2.69E-03	1.061644224	0.941935139	0.676750659
carnitine.C3	2.95E-03	3.13E-03	1.061343401	0.942202118	0.87208868
5-aminolevulinic acid	2.54E-02	2.69E-02	1.059636012	0.943720286	0.475058271
ornithine	1.09E-04	1.16E-04	1.058361534	0.944856713	0.79980025
AMP	3.87E-02	4.07E-02	1.050312824	0.952097296	0.432214187
xanthine	4.76E-04	4.98E-04	1.044976081	0.956959703	0.852547158
indole	3.99E-03	4.14E-03	1.039263538	0.962219844	0.770465212
glyoxylate	4.20E-04	4.36E-04	1.037452839	0.963899237	0.515512348
sedoheptulose-7-phosphate	1.80E-04	1.87E-04	1.037127387	0.96420171	0.854082869
ascorbic acid	7.92E-02	8.20E-02	1.034929442	0.966249446	0.630654744
phosphorylcholine	9.73E-04	1.01E-03	1.033722072	0.967378009	0.673595383
SDMA.ADMA	9.87E-05	1.02E-04	1.029396682	0.971442804	0.862333149
1-methyl-adenosine	4.33E-03	4.45E-03	1.028338173	0.972442749	0.847063439
N-acetyl-alanine	2.79E-02	2.87E-02	1.027739448	0.97300926	0.314168055
S.adenosyl.methionine	4.42E-03	4.55E-03	1.027701851	0.973044856	0.701620695
N.acetyl.aspartic acid	4.22E-03	4.32E-03	1.025243396	0.975378144	0.648846351
glutamate	2.80E-03	2.87E-03	1.024485944	0.976099287	0.479058575
citrulline	6.83E-04	6.98E-04	1.023247628	0.977280545	0.865912743
acetyl.carnitine	1.45E-03	1.48E-03	1.023057876	0.977461807	0.636058203
carbamoylphosphate	6.88E-04	7.04E-04	1.022451148	0.978041838	0.798595663
succinate	9.84E-03	1.00E-02	1.018232016	0.982094439	0.909599721

© 2019. Li W et al. Published online at http://www.aginganddisease.org/EN/10.14336/AD.2019.0120

glycine	2.88E-04	2.92E-04	1.014415978	0.985788889	0.951679438
homocysteine	1.96E-04	1.99E-04	1.012611636	0.987545436	0.88521244
S-adenosyl-homocysteine	1.76E-04	1.78E-04	1.012364906	0.987786117	0.939739388
cADP-ribose	1.17E-02	1.18E-02	1.007705194	0.992353722	0.880341028
a-ketoisovaleric acid	3.45E-03	3.47E-03	1.005128531	0.994897637	0.947429541
y.aminobutyric acid	1.18E-03	1.18E-03	1.004349716	0.995669122	0.972762242
orotate	2.84E-04	2.84E-04	1.000091375	0.999908634	0.998904452
2-isopropylmalic acid	9.96E-02	9.86E-02	0.990143301	1.009954821	0.853527761
NADP	1.15E-03	1.13E-03	0.984389868	1.015857672	0.923542439
putrescine	3.02E-03	2.97E-03	0.984354648	1.015894019	0.915652083
pyroglutamic acid	7.64E-05	7.51E-05	0.982475517	1.017837068	0.796202425
proline	1.27E-02	1.23E-02	0.971457766	1.029380829	0.702285449
alanine	1.80E-03	1.74E-03	0.970996072	1.029870284	0.867016577
isoleucine	7.97E-04	7.73E-04	0.97089319	1.029979415	0.837949479
creatine	5.24E-02	5.05E-02	0.964792742	1.036492043	0.79347777
urea	1.24E-02	1.20E-02	0.963522683	1.037858286	0.674756541
pantothenic acid	7.45E-04	7.17E-04	0.963512614	1.037869132	0.734672276
methionine	8.47E-03	8.14E-03	0.960583284	1.041034147	0.758657738
fumarate	1.68E-03	1.61E-03	0.959586569	1.042115461	0.619648448
aspartate	9.94E-04	9.53E-04	0.959220107	1.042513593	0.762873879
serine	5.60E-03	5.36E-03	0.957685001	1.044184673	0.637645343
S.lactoylglutathione	3.52E-03	3.36E-03	0.956634241	1.045331598	0.93143645
creatinine	8.35E-04	7.97E-04	0.954192948	1.048006069	0.853949113
glucosamine	1.06E-03	1.01E-03	0.952356177	1.050027316	0.588168996
aminoadipic acid	1.29E-03	1.22E-03	0.95147306	1.051001906	0.825179848
pyruvate	1.86E-04	1.76E-04	0.949378729	1.053320418	0.524453013
arginine	2.02E-03	1.91E-03	0.948482861	1.054315308	0.6574485
aconitate	5.36E-04	5.07E-04	0.946532651	1.05648759	0.806214466
riboflavin	4.24E-05	4.01E-05	0.94543205	1.057717474	0.893250684
valine	3.69E-03	3.49E-03	0.944938107	1.05827037	0.492228661
trimethyllysine	3.22E-03	3.03E-03	0.942006646	1.061563636	0.444836335
betaine aldehyde	5.66E-05	5.33E-05	0.941808003	1.061787537	0.776436318
O.acetyl.serine	6.92E-04	6.51E-04	0.94037126	1.063409786	0.504260303
methyl-glyoxal	4.32E-04	4.02E-04	0.930564461	1.074616581	0.277584202
2,3-dihydroxybenzoic acid	2.29E-03	2.13E-03	0.928898747	1.076543598	0.435160493
GSH	2.43E-03	2.26E-03	0.928376073	1.077149691	0.660672765
guanosine	1.38E-02	1.28E-02	0.923372617	1.082986415	0.868169793
hydroxyglutaric acid	3.40E-03	3.14E-03	0.922814475	1.083641433	0.378763825
gluconic acid	3.85E-04	3.54E-04	0.921196549	1.085544666	0.729432972
N-acetyl-ornithine	4.64E-04	4.27E-04	0.920887119	1.085909423	0.721548037
citrate-isocitrate	8.47E-02	7.79E-02	0.919700808	1.087310124	0.44640158
glucose-fructose	1.63E-02	1.49E-02	0.914847752	1.093078054	0.651051329
hydroxy.proline	1.10E-03	1.00E-03	0.911917343	1.096590616	0.464599073

© 2019. Li W et al. Published online at http://www.aginganddisease.org/EN/10.14336/AD.2019.0120

SUPPLEMENTARY DATA

ADP	4.91E-02	4.45E-02	0.906544549	1.10308975	0.634229787
lactate	6.34E-04	5.73E-04	0.903788461	1.106453604	0.618008838
shikimic acid	5.88E-03	5.30E-03	0.899882871	1.111255733	0.608706737
glycerophosphorylcholine	2.83E-03	2.54E-03	0.894806104	1.117560548	0.253696841
dAMP	4.08E-03	3.64E-03	0.891577666	1.121607279	0.19808559
homocysteic acid	7.15E-05	6.32E-05	0.883401314	1.131988355	0.647352234
phenyl.alanine	1.17E-02	1.03E-02	0.881284476	1.134707381	0.273606015
glucarate	8.93E-05	7.86E-05	0.880215873	1.136084944	0.492522028
nicotinate	1.08E-04	9.20E-05	0.852685716	1.172765043	0.05252836
glucosamine.6.phosphate	5.27E-04	4.42E-04	0.838816758	1.192155486	0.487693865
xanthosine	1.00E-04	8.40E-05	0.838283353	1.192914063	0.622751867
guanidoacetic acid	5.90E-03	4.94E-03	0.837579831	1.193916046	0.124890467
NAD	1.44E-04	1.20E-04	0.832261648	1.201545214	0.181423065
tyrosine	1.18E-02	9.59E-03	0.811611104	1.232117199	0.200815982
3-hydroxybutyrate	2.60E-04	2.10E-04	0.809072995	1.235982423	0.537940353
p-hydroxybenzoate	1.80E-04	1.45E-04	0.802373585	1.246302244	0.136377813
malic acid	3.14E-04	2.48E-04	0.791870128	1.262833342	0.001966833
myo.inositol	1.26E-03	9.91E-04	0.784111325	1.275329111	0.021829578
adenine	1.35E-03	1.05E-03	0.77282628	1.293951857	0.226398704
phospho-creatine	8.12E-04	6.27E-04	0.77208261	1.295198192	0.627345915
acetyl.choline	2.33E-02	1.76E-02	0.756005139	1.322742332	0.07965308
pyridoxamine	5.90E-04	4.44E-04	0.752298702	1.32925924	0.500777142
malonate	1.77E-04	1.32E-04	0.744682482	1.342854202	0.074219589
dihydroxyacetone-phosphate	5.63E-04	3.79E-04	0.672859593	1.486194164	0.020590639
thymidine	1.81E-04	9.97E-05	0.550375844	1.816940208	0.070424801
Sample Name	Hippo-ctrl	hippo-drug	Drug/ctrl	Ctrl/drug	t-test

© 2019. Li W et al. Published online at http://www.aginganddisease.org/EN/10.14336/AD.2019.0120
